# Hay Fever

**Published:** 1891-10

**Authors:** 


### HAY FEVER.

Hay fever got its name from the fact that it occurs generally about the time hay is gathered. Recent observations of some careful investigators have shown that there are certain physical peculiarities, connected with the inner bones of the nose, which exist in almost all those who suffer from hay fever, and which may reasonably be regarded as among the causes of the disease. That one of the chief causes of hay fever, if not the chief one, is to be found in the atmosphere has been long recognized. But it is by no means easy to say what is the exact character of this detrimental condition. Competent observers, both in England and America, have made careful studies of this subject and have found many vegetable spores, or microscopical seeds, liberally distributed through the air during the season of the year at which hay fever prevails, and have naturally concluded that there is some connection between the floating seeds and the disesse under consideration. The onset of hay fever is gradual. The first attack consists only of more or less severe catarrh. Often for many years there is no asthma present. With each recurring attack, however, the symptoms become more severe, until at last the climax is reached, and what may be regarded as the habit is fully formed. The effect of what we call habit is further shown by the fact that the susceptibility of the sufferer from hay fever seems to be diminished by several years of exemption, from residence in a suitable locality. Hay fever does not imperil life—it is even laughed at by those who do not have it—but when at all severe it inflicts much suffering, and most materially interferes with most of life's avocations, by obliging the man who has it to give up all engagements, and to practically suspend his business for from four to six weeks in each year.

